# Interfacial charge-mediated non-volatile magnetoelectric coupling in Co_0.3_Fe_0.7_/Ba_0.6_Sr_0.4_TiO_3_/Nb:SrTiO_3_ multiferroic heterostructures

**DOI:** 10.1038/srep07740

**Published:** 2015-01-13

**Authors:** Ziyao Zhou, Brandon M. Howe, Ming Liu, Tianxiang Nan, Xing Chen, Krishnamurthy Mahalingam, Nian X. Sun, Gail J. Brown

**Affiliations:** 1 Department of Electrical and Computer Engineering, Northeastern University, Boston, MA, USA, 02115; 2 Materials and Manufacturing Directorate, Air Force Research Laboratory, Wright-Patterson AFB, OH, USA 45433-7707; 3 Energy Systems Division, Argonne National Laboratory, Lemont, IL, USA, 60439

## Abstract

The central challenge in realizing non-volatile, E-field manipulation of magnetism lies in finding an energy efficient means to switch between the distinct magnetic states in a stable and reversible manner. In this work, we demonstrate using electrical polarization-induced charge screening to change the ground state of magnetic ordering in order to non-volatilely tune magnetic properties in ultra-thin Co_0.3_Fe_0.7_/Ba_0.6_Sr_0.4_TiO_3_/Nb:SrTiO_3_ (001) multiferroic heterostructures. A robust, voltage-induced, non-volatile manipulation of out-of-plane magnetic anisotropy up to 40 Oe is demonstrated and confirmed by ferromagnetic resonance measurements. This discovery provides a framework for realizing charge-sensitive order parameter tuning in ultra-thin multiferroic heterostructures, demonstrating great potential for delivering compact, lightweight, reconfigurable, and energy-efficient electronic devices.

Driven by ever-increasing demands for energy-efficient memory and logic devices, the area of spintronics has focused on the realization of electrostatic control of magnetism in a reversible and stable manner. Multiferroic heterostructures, consisting of distinct ferromagnetic, ferroelectric, and ferroelastic phases, exhibit strong strain-mediated magnetoelectric (ME) coupling resulting in large strain-induced changes in magnetic anisotropy with applied electric fields (E-fields). Materials exhibiting strong ME coupling demonstrate great promise for a wide variety of applications in spintronics and electronic devices^1^,^2^,^3^,^4^,^5^,^6^,^7^,^8^. At the most fundamental level, a single control parameter of voltage-induced piezo-strain, arising from ferroelectrics, is used to manipulate magnetic anisotropy and other strain-sensitive properties in real time, including, but not limited to magnetic anisotropy, ferromagnetic resonance, magnetoresistance, Curie temperature, and Metal-Insulator transition^1^,^2^,^3^,^4^. Therefore, ME-based devices are fast, lightweight, and energy efficient; all unique qualities which exhibit great promise for overcoming intrinsic obstacles in spintronics and reconfigurable electronics. For example, many multiferroic devices driven by E-fields have been designed and developed, including voltage-tunable RF/microwave signal processors^5^,^6^, magnetoelectric random access memories (MERAM)^7^, and voltage-tunable magnetoresistance devices^8^.

Other mechanisms have been explored in magnetoelectric heterostructures in order to control magnetic properties through the application of relatively small E-fields. Two of the most common are strain/stress-mediated^9^,^10^,^11^,^12^,^13^ and charge-mediated^14^,^15^,^16^,^17^,^18^,^19^,^20^,^21^,^22^,^23^,^24^,^25^,^26^,^27^,^28^,^29^,^30^,^31^ ME coupling. The former effect (mentioned above) has been well studied and established. For instance, remarkable E-field-induced effective magnetic anisotropy fields of up to 3500 Oe and magnetoelectric coefficients of 580 Oe · cm/kV have been achieved in Terfenol-D/lead zinc niobate-lead titanate (PZN-PT) (011) composite bilayer structures^9^. However, in these reports, the linear ME coupling effect is exploited to realize volatile tuning of magnetism through the converse magnetoelastic effect. Once the operating field is switched off, the magnetization decays back to the initial state as the piezoelectric-induced strain relaxes in the ferroelectric underlayer.

Recently, a voltage-impulse-induced ferroelastic domain switching was used to realize non-volatile electrical switching of magnetization in multiferroic heterostructures^10^. A stable and reversible 71° and 109° ferroelastic polarization switching occurs in FeCoB/lead magnesium niobate-lead titanate (PMN-PT) (011), contributing to large in-plane strain and resulting in non-volatile voltage-impulse tuning of FMR and manipulation of magnetization direction. Such ferroelastic domain switching covers up to 90% of the entire poled area and is much larger than that observed in (001)-oriented PMN-PT. Alternatively, a voltage-impulse-induced bistable magnetization switching based on E-field induced phase transitions can also be exploited, such as in FeGaB/PZN-PT (011) multiferroic heterostructures^12^. By utilizing the dual E- and H-field tunability, dramatically enhanced FMR tunability ranges up to 13.1 GHz can be achieved, which will greatly satisfy engineering requirements for a variety of microwave applications. Extremely large converse magnetoelectric coupling coefficients of 3850 Oe · cm/kV (ΔH/ΔE) are observed at the voltage-induced phase transition points.

Nevertheless, the realization of commercial devices based on non-volatile tuning of magnetism in multiferroic thin films remains challenging due to significant substrate clamping effects^13^. Regardless of whether E-field-induced linear piezoelastic or ferroelastic domain rotation is utilized, the substrate inhibits any deformations raised from the ferroelectric films due to the enormous volume ratio between the ferromagnetic film and underlying ferroelectric substrate. Interfacial charge screening effects have been proposed to be an ideal way to realize non-volatile electrical control of magnetization in multiferroic thin films, as this effect is independent of strain. This effect was initially predicted by first-principle calculations^21^, then demonstrated in many multiferroic systems including metallic/dielectric/ferroelectric heterostructures: Fe/BaTiO_3_ (BTO)^21^, Ni/BaTiO_3_ (BTO)^22^,^23^, Fe/MgO^24^, CoFe/MgO^25^, Fe/BTO^26^,^27^, NiFe/SrTiO_3_^28^, NiFe/PMN-PT^29^, Co/Gd_x_O^31^, Co/P(VDF-TrFE) (copolymer ferroelectric of 70% vinylidene fluoride with 30% trifluoroethylene)^32^ and Oxides/ferroelectric heterostructures: La_0.8_Sr_0.2_MnO_3_ (LSMO)/Pb(Zr_0.2_Ti_0.8_)TiO_3_ (PZT)^33^,^34^. Using this approach, large voltage-induced magnetic anisotropy changes (17.6 kJ/m^3^) have been achieved^24^. This ME mechanism is not limited by substrate clamping effects and requires only very small voltages (<10 V) to provide sufficient surface charge at the ferro/piezo/dielectric thin film interface to induce a surface anisotropy change. Tsymbal et al. revealed theoretically that the charge effect originates from spin-dependent screening in ferro/piezo/dielectric layers induced by the electric field which leads to notable changes in the surface magnetization and surface magnetocrystalline anisotropy (MAE) in Fe/BaTiO_3_ multiferroic heterostructures. Shortly thereafter, Suzuki et al. observed experimentally large magnetocrystalline anisotropy changes (13.7 kJ/m^3^) in ultra-thin Fe layers (0.45 nm) driven by surface charge screening in the dielectric MgO layer of Fe/MgO heterostructures^24^. Further, Beach et al, demonstrated E-field induced charge effect in metallic/dielectric thin films heterostructure can drive magnetic domain wall (DW) dynamics in a fast and energy efficient manner^30^,^31^. Nevertheless, in many heterostructures, charge-mediated ME coupling still suffers from volatility, therefore limiting its application in a variety of commercial ME-based devices due to strict energy consumption requirements. Ferroelectric layers^29^, however, instead of dielectric layers^28^, introduce a hysteretic behavior that could therefore act as ideal candidates for realizing non-volatile voltage control of magnetization direction in multiferroic heterostructures. In this scenario, the stable and reversible polarization switching in ferroelectric phases may result in non-volatile tuning of magnetic properties in ME heterostructures due to distinctly different ferroelectric ground states. Nan et al. studied co-existence of strain and charge effect ME coupling in metallic magnetic thin film (NiFe)/ferroelectric (PMN-PT) slab multiferroic heterostructures and reversible, non-volatile voltage control of magnetic anisotropy was realized corresponding to a giant effective tunable magnetic field of ~200 Oe^29^. Although improvement of non-volatility and tunability have been achieved in magnetic thin film/ferroelectric slab multiferroic heterostructures, there are still fundamental limits like high voltage consumption (400 V) and existence of strain effects need to be eliminated for charge mediated voltage control of magnetism.

Ba_x_Sr_1−x_TiO_3_ (BSTO) is a well-known ferro/dielectric material and has attracted much interest for applications including capacitors, high capacity charge storage, and ferroelectric random access memory (due to its high dielectric constant)^35^,^36^. Additionally, BSTO has high permittivity, low loss tangent, and high power handling capability, and is therefore seen as a very promising replacement for conventional ferrite and semiconductor device phase shifters. In this experiment, we intend to use a high K material (BSTO), rather than ferroelectric materials, to accumulate charge for achieving interfacial charge-effect-induced changes in magnetic properties of a thin ferromagnetic overlayer. Most ferroelectric materials possess low symmetric crystal structures, which usually results in a multiple ferroelectric domain structure. Upon applying a voltage, the ferroelectric domain switching may be accompanied by a ferroelastic strain^10^. In this case, the charge and the strain effect would both contribute to the ME effect. However, BSTO with a composition ratio of 6:4 for Ba and Sr, exhibits paraelectric behaviors with high dielectric constant at room temperature. Therefore, the strain effect is neglectable and the charge accumulation would be enhanced dramatically by applying a voltage on a BSTO sample, in comparison to other dielectric substrates such as STO or MgO. Thus, BSTO would be the best candidate for investigating interfacial charge-effect-induced ME coupling, without the influence of ferroelastic strain^24^,^28^.

Therefore, we fabricate CoFe (1.2 nm)/BSTO (Ba_0.6_Sr_0.4_TiO_3_) (~600 nm) multiferroic heterostructures on 0.5 mm-thick 0.7 wt.% Nb-doped SrTiO_3_ (001) (Nb:STO) substrates by pulsed laser deposition (PLD) and subsequent ex-situ sputter deposition of CoFe. Resultant heterostructures are then systematically investigated by ferromagnetic resonance (FMR) measurements under various applied out-of-plane (OOP) voltages, from 10 V to −10 V, across the BSTO layer. FMR measurements are taken at different applied magnetic field angles in the plane of the CoFe thin film. Ultimately, a giant FMR field shift of ~40 Oe, driven by voltage impulses (<100 ms) is achieved in the CoFe/BSTO bilayer structure, corresponding to an out-of-plane anisotropy change of 7 kJ/m^3^, and confirmed by 360° angular-dependent FMR measurements. The measured FMR field shift is robust, non-volatile, and repeatable, allowing voltage impulses, instead of a constant voltage, to manipulate magnetism. This progress alleviates substrate clamping effects and offers a much more energy-efficient tuning mechanism in spintronics devices such as electric field writing in random access memory (MERAM) devices.

## Results

**Figure 1:**
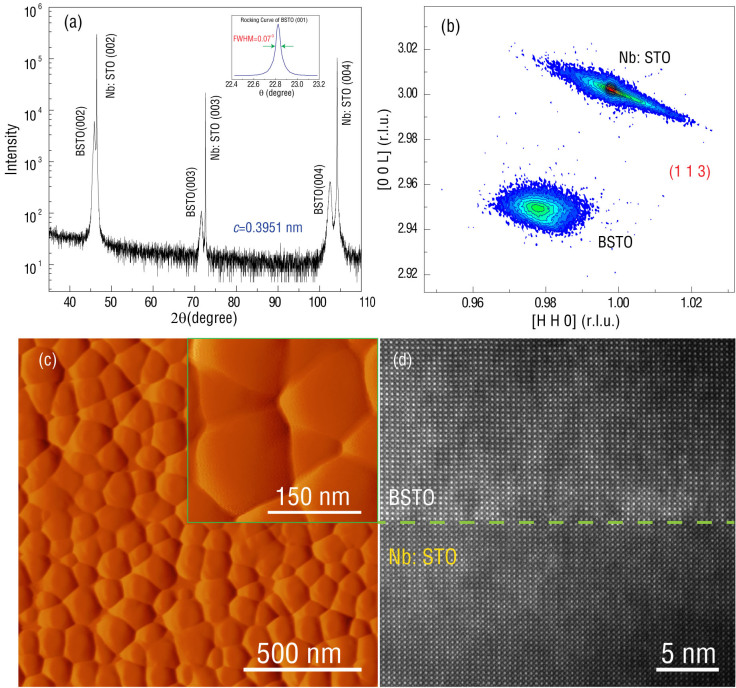
Microstructural and Surface Morphological Study of BaSrTiO_3_ Nb:SrTiO_3_ (001). (a) Typical High-Resolution X-ray Diffractogram (XRD) of BaSrTiO_3_/Nb:SrTiO_3_ (001); (b) High-Resolution Reciprocal Lattice Map about (113) reflections from both film (BSTO) and substrate (Nb:STO); (c) Atomic Force Micrograph (AFM) of the BSTO surface; (d) High-Resolution Transmission Electron Micrograph (HR-TEM) image of the BaSrTiO_3_/Nb:SrTiO_3_ interface. This series of results demonstrate the high-quality, epitaxial nature of the BaSrTiO_3_/Nb:SrTiO_3_ layers produced during this study.

CoFe/BSTO heterostructures (~600 nm BSTO layer) are grown on (001)-oriented Nb:SrTiO substrates by PLD. Microstructural and surface morphological studies are summarized in Figure 1. A typical high-resolution x-ray diffractogram (coupled ω-2θ scan) is presented in Figure 1(a). The only observable reflections are those allowed by the film and substrate. The rocking curve about the (001) BSTO peak (θ = 22.8°) has a full width at half maximum (FWHM) equal to 0.7°. The lattice parameter of the BSTO layer is 0.3951 nm accordingly. Figure 1(b) is a typical high-resolution reciprocal lattice map (HR-RLM) about (113) reflections from the film (BSTO) and substrate (Nb:STO (001)), showing that films are fully relaxed and epitaxial in nature. The surface morphology of BSTO was examined by atomic force microscopy (AFM) and presented in Figure 1(c). A well-defined smooth BSTO surface was observed with large (100 ~ 200 nm) domains. The interface between BSTO layer and Nb:STO substrate, as well as the overall crystallinity, was examined by high resolution transmission electron microscopy (HR-TEM), as shown in Fig. 1(d). The atomic column resolution and abrupt interface evident in this micrograph, coupled with the above results, demonstrate the high quality and epitaxial nature of BSTO films produced during this study.

**Figure 2:**
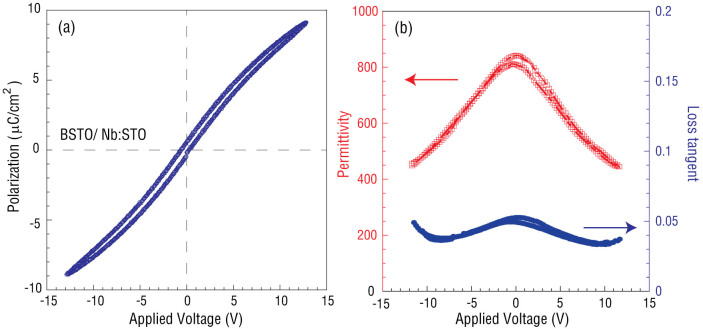
Electrical properties of BSTO layer. (a) Polarization vs applied voltage of BSTO layer; (b) Permittivity and loss tangent vs applied voltage of BSTO layer.

Figure 2(a) and (b) represent the polarization; permittivity and loss tangent of BSTO layer, respectively, depending on the applied voltage cross the film. BSTO films demonstrate high permittivity (~800) with small tangent loss (<0.05); ideal electrical properties of BSTO for voltage control.

**Figure 3:**
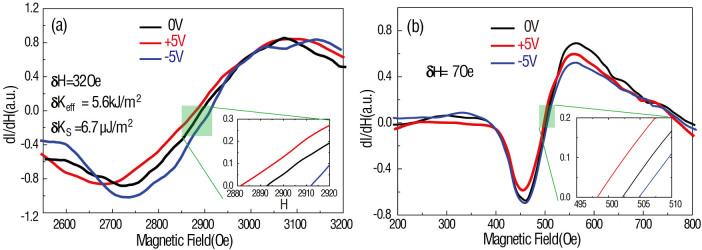
Ferromagnetic resonance spectra of CoFe/BSTO multiferroic heterostructure under varied applied voltage. (a) FMR spectra of CoFe (1.2 nm)/BSTO (600 nm) multiferroics heterostructure under different applied voltages, 0 V, −5 V, 5 V; (b) FMR spectra of CoFe (50 nm)/BSTO (600 nm) multiferroics heterostructure under different applied voltages, 0 V, −5 V, 5 V.

For this study, 1.2 nm and 50 nm CoFe thin films are deposited through a shadow mask with 400 μm diameter through holes on top of the BSTO/Nb:STO (001) layers using DC magnetron sputter deposition. The E-field control of magnetic anisotropy of CoFe (1.2 nm)/BSTO (600 nm) heterostructures is determined by FMR measurements, as in Figure 3(a). We choose 1.2 nm CoFe thin film because the charge effect strength is highly dependent on magnetic film thickness^24^,^28^ and we obtained the maximum charge effect strength at CoFe thickness around 1.2 nm in our previous research on CoFe/STO heterostructures^28^. As previously pointed out, the charge effect can only exist in ultra-thin (~1 nm) magnetic films due to the Thomas-Fermi screening effect. As the magnetic film thickness increases to ≫1 nm, the charge effect disappears rapidly^24^,^25^,^28^,^29^. For example, the charge effect disappears at magnetic layers ≫1 nm in many magnetic/dielectric heterostructures, such as Fe/MgO^24^, Co_20_Fe_80_/MgO; in magnetic/MgO systems, it was found that a very strong charge screening effect occurs when the thickness of Fe thin film was 0.45 nm (ref. 24). Nevertheless, in the multiferroics heterostructures of CoFe/SrTiO_3_ (STO)^28^ and NiFe/PMN-PT^29^, strong charge effects were observed in the 1 nm thickness range of the magnetic layers. As the magnetic layer is deposited onto a relative rough surface, like STO^28^, PMN-PT^29^ and BSTO (see Fig. 1(c), AFM image) in this paper, it requires thicker magnetic layer to form a continuous film. Therefore, the critical thickness (where the maximum charge effect appears) could be larger compared to others' result^24^,^25^. For comparison, voltage control of 50 nm-thick CoFe on epitaxial BSTO (600 nm) is also measured and shown in Fig. 3(b). While there could be a co-existence of strain and charge mediated magnetoelectric coupling in CoFe (1.2 nm)/BSTO heterostructures, CoFe (50 nm)/BSTO heterostructures will only demonstrate strain-mediated magnetoelectric coupling due to the non-existence of charge-mediated magnetoelectric coupling in relatively thicker magnetic films (≫1 nm). By studying the ME coupling-induced FMR field change in both heterostructures, we can distinguish between the strain- and charge-mediated ME coupling mechanisms in CoFe (1.2 nm)/BSTO heterostructures^28^,^29^. Figure 3(a) shows typical FMR spectra from CoFe (1.2 nm)/BSTO (600 nm) layers with applied voltages varying from −10 V to 10 V across BSTO layer. To apply voltages >10 V would cause a leakage problem for 400 μm diameter CoFe top electrodes, which is required in FMR measurements due to the sensitivity of ESR system; the ESR system can barely detect CoFe dots with diameter smaller than 400 μm. For P(E) loop measurements, the applied voltage can be larger than 10 V due to smaller top electrode with diameter around 50 μm. The FMR field, which is defined as the zero-cross of the FMR spectrum, is shifted from 2881 Oe to 2913 Oe with the gap of 32 Oe, shown in the inset. Figure 3(b) demonstrates voltage control of FMR in CoFe (50 nm)/BSTO (600 nm) heterostructures, and in comparison, there is no significant FMR field shift (<10 Oe) in thicker CoFe films. Based on our analysis, the strain-mediated ME coupling strength is very weak in CoFe/BSTO heterostructures, while the interfacial charge effect-mediated ME coupling dominates FMR field changes, or in other words, the effective magnetic anisotropy field change.

**Figure 4:**
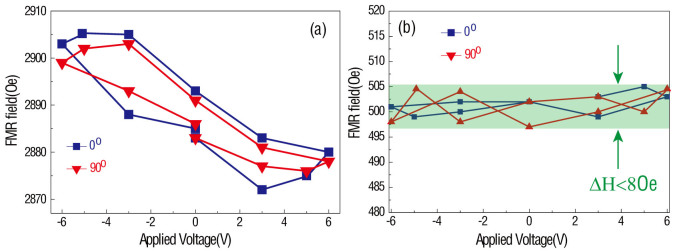
Ferromagnetic resonance field vs. applied voltage in CoFe/BSTO heterostructure with different CoFe thickness. (a) FMR field dependence of applied voltage measured along different applied magnetic field orientations of CoFe (1.2 nm)/BSTO (600 nm) multiferroics heterostructure; (b) CoFe (50 nm)/BSTO (600 nm) multiferroics heterostructure.

Furthermore, the FMR field dependence on various applied voltages is summarized in Figure 4(a) and (b) for CoFe (1.2 nm)/BSTO (600 nm) structures, and reveals a hysteresis loop-like behavior. On one hand, Figure 4(a) has great similarity with Figure 2(a); the P(E) loop of the BSTO layer. On the other hand Figure 4(b), relatively speaking, demonstrates a random dependence on applied voltage. This behavior infers that the FMR field change is induced by surface charge. For instance, the charge accumulation at the interface between ferro/piezo/dielectric and ferromagnetic thin films will alter the ground state of the magnetic thin film thereby changing the surface anisotropy, thus changing the total magnetic anisotropy in the magnetic film^21^,^24^. The charge-mediated ME coupling strength is therefore proportional to the surface charge accumulation, or rather, the polarization of the BSTO layer^21^,^22^,^23^,^24^,^25^,^26^,^27^,^28^,^29^.

## Discussion

To simulate the magnetic anisotropy change quantitively, the total energy of CoFe film can be represented as (equation (1))^24^: 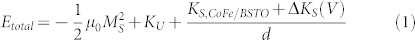
Where, E_total_ is the total energy of CoFe thin film, M_S_ is saturation magnetization, K_U_ is bulk anisotropy, K_S_ is surface anisotropy between CoFe and BSTO layer, and ΔK_S_(V) is the charge-effect-induced surface anisotropy change. The calculated FMR frequency corresponding to this is (equation (2)): 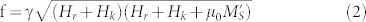
in which H_r_ is the FMR field, and H_k_ is the bulk anisotropy field (in our case, 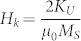, due to isotropic behavior). M_S_′ is effective saturation magnetization, where, 
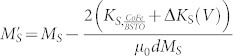
. All measurements were taken at a resonance frequency of 9.55 GHz; H_r_ = 500 Oe for the 50 nm-thick CoFe film, and H_r_ = 2890 Oe for 1.2 nm CoFe film. Considering the FMR field equation of d = 1.2 nm and 50 nm without applied voltages (ΔK_S_(V) = 0 μJ/m^2^), K_S,CoFe/BSTO_ = 4.2 mJ/m^2^ (with M_S_ = 2.23 Tesla). The effective anisotropy change (K_eff_ = ΔHM_S_ = 5.6 kJ/m^3^) and corresponding surface anisotropy change (ΔK_S_ = ΔK_eff_d = 6.7 μJ/m^2^) are obtained. Our result is comparable to Suzuki's Fe/MgO, with ΔK_S_ = 8.4 μJ/m^2^ 24. The smaller anisotropy change in our CoFe/BSTO heterostructure may due to the slight surface roughness of the BSTO layer, therefore limiting the overall strength of the surface charge effect.

**Figure 5:**
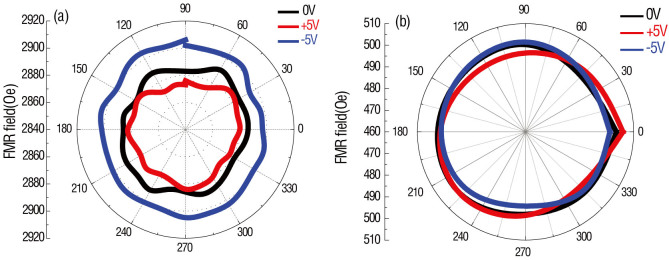
Angular study of Ferromagnetic resonance field vs. applied voltage in CoFe/BSTO heterostructure with different CoFe thickness. (a) Angular dependence of FMR field in CoFe (1.2 nm)/BSTO (600 nm) multiferroic heterostructures measured at different voltages, from 5 V to −5 V; (b) angular dependence of FMR field in CoFe (50 nm)/BSTO (600 nm) multiferroics heterostructure measured at different voltage, from 5 V to −5 V.

To ensure the induced interfacial charge is the dominant effect driving the change in magnetic anisotropy, angular dependence of the voltage-induced FMR field in CoFe/BSTO heterostructures with different CoFe layer thicknesses, 1.2 nm and 50 nm, are investigated. In theory and experiment, the charge effect provides an out-of-plane magnetic anisotropy change resulting in an isotropic in-plane magnetic anisotropy change due to the isotropic nature of the charge distribution in-plane^21^,^22^,^23^,^24^,^25^,^26^,^27^,^28^,^29^, which means the FMR field shifts measured along different magnetic field directions are essentially equivalent. In contrast, the strain-mediated ME coupling provided by epitaxial ferroelectric thin films is anisotropic in-plane due to the highly anisotropic strain distribution inherent in the ferroelectric thin film^9^,^10^,^29^. If the strain/stress effect dominates, the FMR field shifts measured along different magnetic field directions would also be different. As shown in Figure 5(a), isotropic FMR field shifts occurring under various applied voltages (−5 V, 0 V, 5 V) are observed. This result demonstrates that there exists no clear in-plane magnetic anisotropy change in the CoFe film and the FMR field shift must be induced by an out-of-plane magnetic anisotropy change. In Figure 5(b), there is no significant FMR field shift in the 50 nm CoFe film. This observation provides solid evidence that the in-plane strain/stress effect does not contribute to voltage-induced magnetic anisotropy changes and that the isotropic surface magnetic anisotropy change is only induced by interfacial charge between CoFe and BSTO.

In commercial E-field tunable magnetics applications, voltage impulses have many advantages compared to having to maintain a constant field. Firstly, voltage impulses require much less energy than maintaining a constant voltage. Secondly, it is much easier to generate voltage impulses in integrated circuits. Many studies have been made on a variety of magnetic/dielectric heterostructures^21^,^22^,^23^,^24^,^25^,^26^,^27^. Although a significant magnetic anisotropy change (17.6 kJ/m^3^) can be obtained in these heterostructures^24^, the anisotropy change is electrically volatile, due to the linear dependence between polarization of the dielectric thin film with applied voltages. To realize non-volatile voltage control, ferroelectric layers with remnant polarization states should be considered. In our previous research regarding ultra-thin NiFe (1.5 nm) on (011)-oriented PMN-PT multiferroic heterostructures, an effective magnetic field of 202 Oe can be obtained during non-volatile switching due to the co-existence of strain- and charge-mediated ME coupling when driven by ~400 V. In real ME applications, small operational voltages (~10 V) are required. Therefore, ferroelectric thin films instead of ferroelectric substrates, manipulated by ~10 V external voltages, could potentially lead to non-volatile functionality ME devices. In our study, the BSTO layer has ferroelectric properties, displaying a polarization hysteresis loop (as shown in Figure 2(a)). Due to the existence of remnant polarization states within BSTO, non-volatile switching could be established.

**Figure 6:**
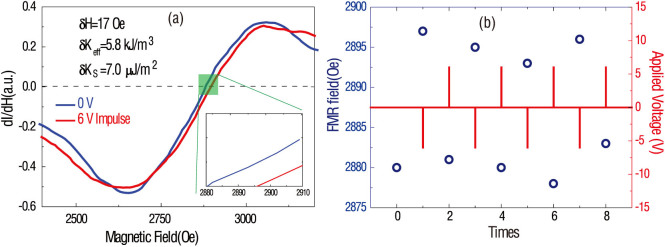
Non-volatile voltage control of FMR field in CoFe/BSTO heterostructure. (a) FMR spectra measured before and after voltage impulse (6 V, <100 ms); (b) Voltage impulses (6 V, −6 V, <100 ms) induced non-volatile FMR field shift.

In Figure 6(a), a voltage impulse with amplitude of 6 V (<100 ms) is applied to the BSTO layer. A non-volatile FMR field switch of 17 Oe occurs (after 100 cycles, we can still obtain ~15 Oe FMR field shift, showing a good repeatability), representing an effective anisotropy change of ΔK_eff_ = 3.0 kJ/m^3^, and surface anisotropy change of ΔK_S_ = 3.6 μJ/m^2^. By applying alternating voltage impulses (6 V, −6 V), as shown in Figure 6(b), the FMR field can be reliably switched back and forth from ~2880 Oe to ~2897 Oe. Here, the capacitor effect plays an important role. Charge remains on in the electrode without depletion after switching the voltage impulses, therefore, the FMR fields remain until the next voltage impulse occurs, due to remnant charge effect. Therefore, in CoFe/BSTO multiferroic thin films heterostructures, the magnetic and RF/microwave properties can be manipulated by smaller amplitude voltage impulses, and with repeatability. Our progress provides a viable route towards realizing non-volatile control of magnetism through E-field induced surface charge accumulation.

## Conclusions

In summary, we have demonstrated a reversible and non-volatile switching of magnetism through application of small electric fields (~10 V) by way of charge-mediated magnetoelectric coupling in CoFe/BSTO/Nb:STO (001) heterostructures. Voltage control of ferromagnetic resonance within multiferroic heterostructures can be utilized to investigate the charge-mediated magnetoelectric behavior quantitatively. An effective magnetic field change of 40 Oe is observed in CoFe/BSTO/Nb:STO (001) heterostructures via surface charge-mediated magnetoelectric coupling. Reversible non-volatile effective magnetic field tuning ranges of 17 Oe are obtained due to different remnant polarization states within the BSTO layer. Finally, charge-mediated magnetoelectric coupling and nonvolatile switching between remnant polarization states in magnetic/ferroelectric thin film heterostructures could lead to next generation of non-volatile magnetoelectric devices.

## Methods

600 nm Ba_0.6_Sr_0.4_TiO_3_ (BSTO) thin films are epitaxially deposited on Nb:SrTiO_3_ substrates using pulsed laser deposition (PLD) using an excimer laser with wavelength 248 nm. Samples are first ultrasonically cleaned in successive rinses of acetone and ethanol before being blown dry with dry N_2_, then introduced into a load-lock with a base pressure of <10^−7^ Torr before being transferred to the growth chamber with a base pressure of <10^−9^ Torr. The substrates are then heated up to 650°C for one hour before depositing at 1 Hz repetition rate under 200 mTorr oxygen, and post annealed for one hour under the same temperature and pressure. Samples are then removed from the chamber and introduced into a separate, magnetron sputter deposition for magnetic film growth. The deposition rate is measured using a quartz crystal thickness monitor (QCM), found to be 0.035, 0.045 Å/sec with a drift of 0.5% per hour. 1.2 nm CoFe thin films are grown on BSTO layers by sputtering method at 10^−7^ pressure using a shadow mask with 400 μm diameter through-holes. High-resolution transmission electron microscopy (HR-TEM) and high-resolution reciprocal space mapping (RSM) are used to characterize the microstructure and crystallinity of the heterostructures. Surface morphology and ferroelectric properties are examined using atomic force microscopy (AFM) and piezo-response force microscopy (PFM), respectively. FMR measurements are taken using an electron spin resonance (ESR) system.

## Author Contributions

Z.Z. and B.H. have equally contribution to this work with preparation and characterization of the samples. M.L., N.S. and G.B. initiated the original idea. Z.Z., B.H. and M.L. wrote the manuscript. B.H. and Z.Z. did the thin film deposition process and Z.Z. did the main experiments with T.N.'s assistant. X.C. discussed the data. K.M. did HRTEM images.
